# Rapid and accurate *in silico* solubility screening of a monoclonal antibody library

**DOI:** 10.1038/s41598-017-07800-w

**Published:** 2017-08-15

**Authors:** Pietro Sormanni, Leanne Amery, Sofia Ekizoglou, Michele Vendruscolo, Bojana Popovic

**Affiliations:** 1 Department of Chemistry, University of Cambridge, Cambridge, CB2 1EW UK; 2 Biopharmaceutical Development, Medimmune Ltd, Granta Park, Cambridge, CB21 6GH UK; 3 Antibody Discovery and Protein Engineering, Medimmune Ltd, Granta Park, Cambridge, CB21 6GH UK

## Abstract

Antibodies represent essential tools in research and diagnostics and are rapidly growing in importance as therapeutics. Commonly used methods to obtain novel antibodies typically yield several candidates capable of engaging a given target. The development steps that follow, however, are usually performed with only one or few candidates since they can be resource demanding, thereby increasing the risk of failure of the overall antibody discovery program. In particular, insufficient solubility, which may lead to aggregation under typical storage conditions, often hinders the ability of a candidate antibody to be developed and manufactured. Here we show that the selection of soluble lead antibodies from an initial library screening can be greatly facilitated by a fast computational prediction of solubility that requires only the amino acid sequence as input. We quantitatively validate this approach on a panel of nine distinct monoclonal antibodies targeting nerve growth factor (NGF), for which we compare the predicted and measured solubilities finding a very close match, and we further benchmark our predictions with published experimental data on aggregation hotspots and solubility of mutational variants of one of these antibodies.

## Introduction

Owing to their high affinity and specificity, as well as their inherently low toxicity, biologics, and in particular monoclonal antibodies, represent the fastest growing class of therapeutic molecules in the biopharmaceutical market^1–3^. Antibodies are currently used as therapeutics across diverse clinical settings, including oncology, chronic inflammatory diseases, infectious diseases and cardiovascular medicine^4^. For most targets of interest, novel antibodies can be obtained through the laboratory screening of large number of variants produced by library construction or by immunization techniques^2, 3^. These screening procedures, including phage display and related techniques, generally yield many candidates binding to a given target with good affinity, and only the tightest binders are usually taken forward for further characterization and development. The main reason behind this choice is that the steps following antibody discovery, such as development of a cell line, optimization of the manufacturing process and of the formulation, are highly resource intensive and therefore can typically be carried out for only one or very few candidates^5^.

In addition to its activity, a key property of a promising drug candidate is its ‘developability’ - the likelihood of its successful development into a stable, safe, and effective drug^6^. For example, if an antibody is to serve a therapeutic purpose, it usually needs to be formulated at the high concentrations necessary for subcutaneous delivery (>50 mg/mL), and it must remain active over the shelf-life of the product (>1 year)^7^. Because of these requirements, a bottleneck for the successful development of antibody therapeutics is often insufficient solubility, which can lead to aggregation at the conditions of storage^8–10^. Aggregation may render an antibody non-functional, not to mention that some protein aggregates can also be toxic or may elicit an immune response in the patients, so that even a small fraction of aggregated proteins can jeopardise the success of a clinical trial^11^. In many instances, insufficient solubility is the main reason behind the high development costs of antibodies^9^.

Traditionally, developability assessments are performed quite late in the antibody-discovery pipeline, which can lead to the failure of some lead antibodies that have been through comprehensive *in vitro* characterization, and have sometimes already undergone testing in animal models or in cultured cells^6^. The practice of assessing developability late is mostly caused by the fact that a large amount of data collected over a large period of time is needed to estimate the aggregation propensity at the intended storage conditions^10^. Furthermore, other measurements of solubility in industry usually need to be done in a rapid manner with limited amount of material, which can make the outcome non-quantitative and often non-reflective of the long-term, high-concentration, stability properties of the antibody under consideration^12^.

**Figure 1:**
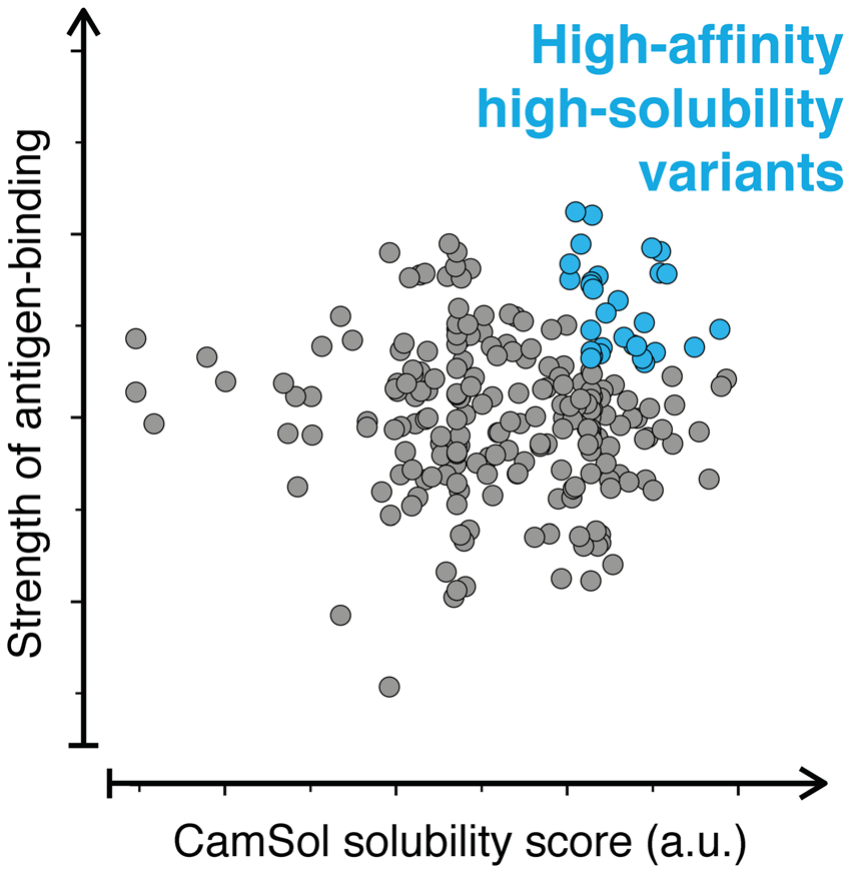
Simultaneous screening of affinity and solubility of antibody libraries. The screening of the antibodies derived from an *in vitro* discovery experiment (e.g. phage display) can be performed using two parameters: (1) a measured binding strength (e.g. binding affinity or off-rate), and (2) a predicted solubility score (e.g. the CamSol intrinsic solubility; a.u. stands for arbitrary units as the scores are dimensionless). The latter is readily computed from the amino acid sequence, thus enabling the selection from the initial screening of lead antibodies with high affinity and solubility.

In order to reduce time and costs, and more importantly to maximize the probability of success of those antibodies selected as leads after library screening, it is increasingly recognized that developability assessments should be performed earlier, ideally during the lead-selection process^6, 13^. In particular, it would be desirable to introduce a reliable assessment of solubility early on at the antibody-discovery stage. To be effective this assessment should be rapid, inexpensive (in particular in terms of material needed), and readily applicable to most, if not all, of the screened antibodies, so that those antibodies embodying the best balance between strength of target binding and solubility can be selected from the very beginning (Figure 1). Given these requirements and the large number of antibody variants that typically result from the screening (up to thousands), it would be particularly convenient to do this early assessment computationally. Computational approaches are in fact already increasingly employed at this stage, for instance to identify potential sequence liabilities, which could impact on deamidation or other possible sources of instability (e.g. oxidation, fragmentation, etc.)^13–18^.

As antibodies in general, and in particular those screened from a given laboratory-constructed library, have a high degree of sequence similarity, methods of predicting solubility changes upon mutation are particularly well-suited for this purpose. In this context, the CamSol method of predicting solubility changes upon mutations, and in particular the sequence-based intrinsic solubility score, may be particularly helpful^19^. To test this strategy, in this work we perform a quantitative validation of the ability of CamSol to predict, from the amino acid sequence alone, the solubility of a number of pharmaceutically-relevant monoclonal antibodies selected from phage or ribosome display^20–22^.

**Figure 2:**
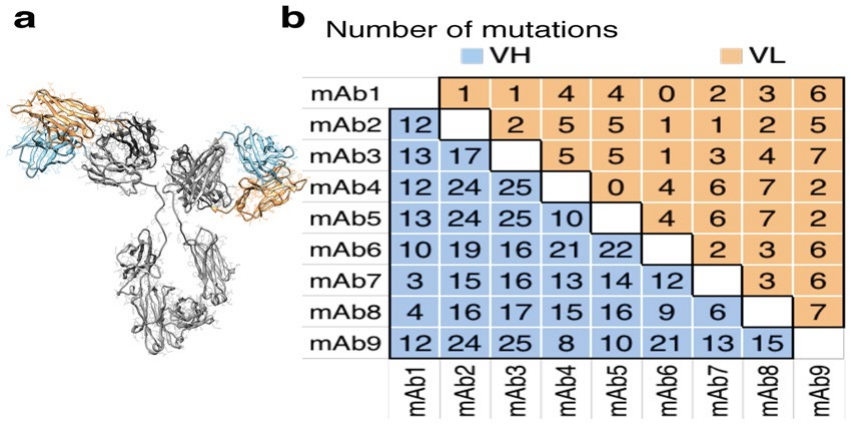
Comparison of the monoclonal antibody variants used in this study. (**a**) Structural model of a monoclonal antibody with the constant domains coloured in grey and the variable domains in orange (VL) and light blue (VH). (**b**) Table summarising the number of mutations distinguishing any two mAb variants employed in this study; the VH domain is shown in the lower-left half and the VL domain in the upper-right half.

Previous applications of the CamSol method have included for example the modulation of the solubility of a single-domain antibody with a very strong correlation between predictions and experiments^19^, or the rational design of the solubility of an immunoglobulin domain (β2-microglobulin) without affecting its stability^23^. However, the protein-variants employed in those studies were small globular proteins differing by at most three residues, so the question of whether CamSol can effectively predict the solubility of large multi-domain protein complexes differing by several mutations, such as monoclonal antibodies obtained from the screening of a phage-display library, is still open. In the current study, we use CamSol to predict the solubility of full-length monoclonal antibodies differing by up to 32 residues in their variable domains (Figure 2, minimum difference 5, median difference 19 residues). Our results show that predicted and measured solubility values are highly correlated, thus demonstrating that the CamSol method can be highly effective in identifying the most soluble lead antibodies just after the sequencing of the screened library. The ‘two-dimensional’ screening strategy that we propose here (Figure 1) thus offers a powerful alternative to experimental approaches for the early assessment of developability^6^, as CamSol can screen thousands of sequences in less than a minute on a standard laptop.

## Results

### mAbs panel

In this study, we employ nine different monoclonal antibodies (referred to as mAbs1 to mAbs9) that bind nerve growth factor (NGF), which were generated from phage display followed by targeted mutagenesis of a parent antibody^21, 22^ (mAb1). Since display and screening were carried out with single-chain fragments, all mAb variants are identical in their constant domains, with all mutations located in the variable domains (VH and VL). The total number of mutations between any two mAbs varies from 5 (mAb1 & mAb7) to 32 point-mutations (mAb3 & mAb9), with a median number of 19 (Figures 2 and S1).

### Experimental characterization of the mAbs

**Figure 3:**
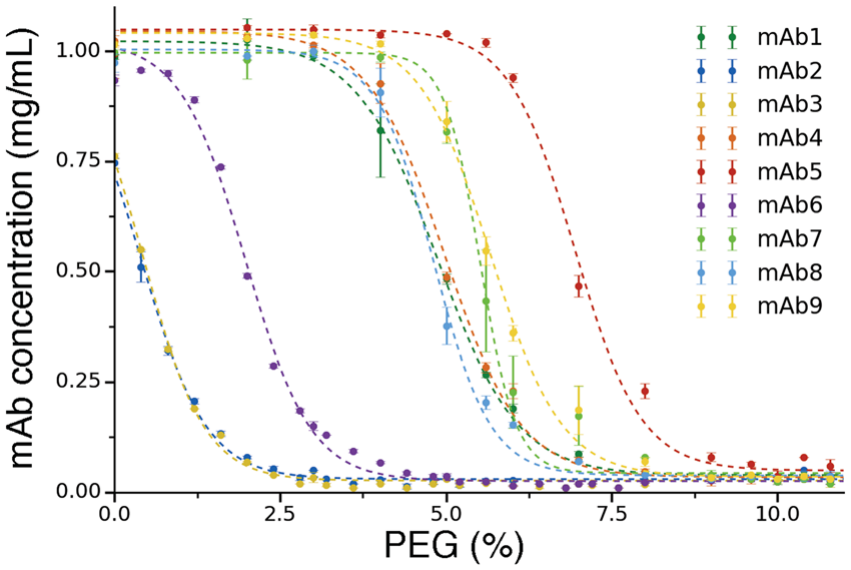
PEG-precipitation assay on the 9 mAbs targeting NGF analysed in this work. Plot of soluble mAb concentrations (y-axis) versus percent PEG amounts (weight/volume, x-axis). Data points are fitted with a sigmoidal curve (broken lines) and the value of the fitting parameter *PEG*_*1/2*_ is used as a proxy for the solubility (see Methods and Figure S2).

The solubility of the different monoclonal antibodies was measured with a PEG-precipitation assay (see Methods). The average soluble protein concentrations obtained from triplicate A280 measurements were plotted against percent PEG (weight/volume) for each of the mAbs tested (Figure 3). Data points were fitted with a sigmoidal curve (see Methods) to estimate *PEG*_*1/2*_, which is defined as the percentage of PEG at which the soluble antibody concentration is half of the starting one (Table 1).

**Table 1:** Solubility scores, solubility and stability measurements for all mAb variants studied in this work.

Variant	Solubility measurement		Stability measurement		CamSol intrinsic score (a.u.)		
	PEG_1/2_ (%)	Apparent solubility (mg/mL)	T_m1_ (°C)	T_H_ (°C)	VH	VL	Combined
*mAb1*	4.9 in (4.5,5.1)	18.8 in (9.7,36.9)	65.9	59.8	0.383	0.696	0.025
*mAb2*	0.4 in (0.32,0.47)	0.78 in (0.7,0.86)	55	50.6	−0.304	0.708	−0.465
*mAb3*	0.44 in (0.41,0.48)	0.89 in (0.78,1.01)	54.8	51.3	0.287	0.751	−0.008
*mAb4*	5 in (4.8,5.1)	29.3 in (12.5,68.3)	55.3	51.5	0.42	0.645	0.017
*mAb5*	6.9 in (6.8,7.2)	78.2 in (27.6,207.8)	55.4	50	0.488	0.645	0.067
*mAb6*	2 in (1.9,2)	2.9 in (2.2,3.7)	54.1	48.7	0.007	0.696	−0.248
*mAb7*	5.5 in (5.3,5.8)	23.3 in (5.8,97.1)	55.3	51.6	0.38	0.775	0.076
*mAb8*	4.8 in (4.6,5)	27.2 in (16.5,44)	55.5	51.5	0.282	0.557	−0.142
*mAb9*	5.7 in (5.5,5.8)	50.6 in (26.9,94.7)	55.8	51.3	0.378	0.584	−0.053

*PEG*_*1/2*_ and the apparent solubility are estimated from the fitting of the data points in Figures 3 and S2, respectively. Numbers in brackets are the lower and upper 95% confidence interval calculated with 10^4^ bootstrap cycles. Stability measurements T_m1_ and T_H_ have been performed respectively with DSC and DSF (see Methods). The CamSol scores are reported for the sequences of the VH domain, the VL domain, and for the combined VH-VL complex (see Methods).

For each of the mAbs, in addition to *PEG*_*1/2*_, we also evaluated the apparent solubility, which is often used in the literature as an estimate of the solubility^24, 25^. To this end, data points in the linear region between the upper and lower plateaus of the sigmoidal curves were replotted on a log scale against percent-PEG on a linear scale (Figure S2). A linear fit was applied and extrapolated back to the y-intercept to determine the apparent solubility. However, in particular for the most soluble mAbs, this extrapolation is affected by large errors, as tiny displacements in the data points in the linear range can cause large differences in the value of the y-intercept in the log-scale (Figure S2a). Thus, in this study we employed *PEG*_*1/2*_ as a proxy for the solubility of the mAb variants, which in our case is strongly correlated with the extrapolated apparent solubility (Pearson’s coefficient of correlation R~0.99, Figure S2b). It is worth noting that the apparent solubility extrapolated from a PEG-precipitation assay may correspond to the thermodynamic solubility only in the case of an ideal system with individual, well-defined, soluble and insoluble states^26^ (respectively the liquid phase and the solid phase). Protein molecules, however, populate a variety of states along their aggregation pathway (including monomers, dimers, oligomers and large aggregates) thus complicating the definition of protein solubility as a single measurable quantity. This problem is particularly present in estimating absolute solubility values, but much less in screening studies such as the present one, as we are interested in solubility differences among the mAb variants, and we operationally define ‘soluble’ those species able to pass through a 0.2 μm filter (see Methods).

The stability of the nine mAbs was also measured by differential scanning calorimetry (DSC) and differential scanning fluorimetry (DSF) (Table 1). We found that all antibodies have melting temperatures of the first transition (T_m1_) above 50 °C and temperature of hydrophobicity^27^ (T_H_) above 48 °C, suggesting that the stability of their native states is not a key determinant of their solubility at the conditions at which solubility measurements were carried out (see Methods).

### *In silico* solubility screening of the mAbs

In order to illustrate the use of the CamSol method for the solubility screening of antibody libraries, we applied it to the nine mAbs for which we measured experimentally the solubility. As the constant domains are the same in all variants, and we are interested here only in solubility differences, we have used as input only the sequences of the VH and VL domains. We calculated the CamSol intrinsic solubility scores for the individual domains as well as for the combined-chains (see Methods and Table 1).

**Figure 4:**
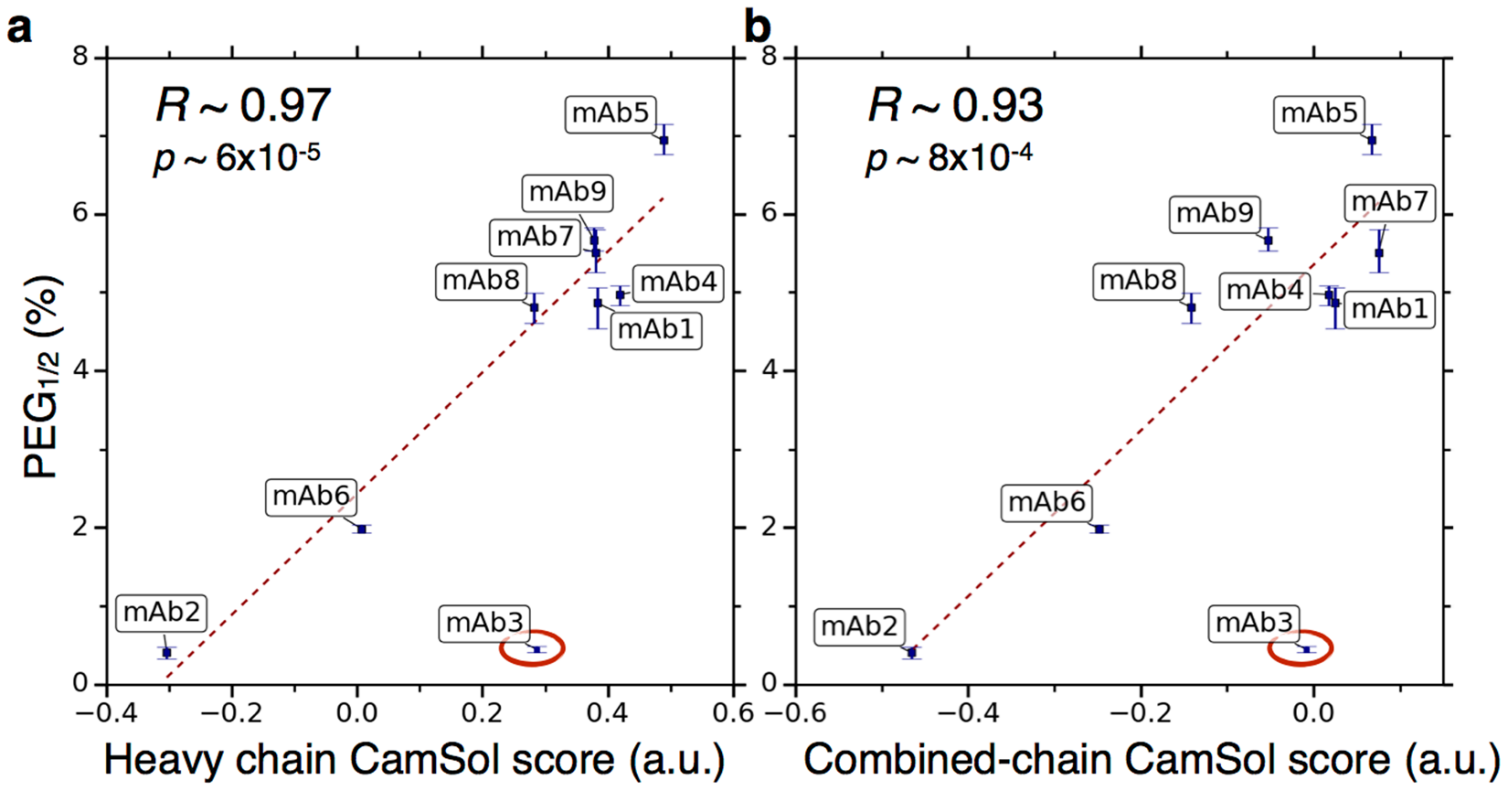
Correlation between the measured solubility and corresponding sequence-based computationally predicted value. Scatter plots of the observed *PEG*_*1/2*_ as a function of the intrinsic CamSol solubility score. Panel (**a**) reports the CamSol scores of the VH domain only, as most mutations are found here (see Figure 2). Panel (**b**) reports a combined score reflecting both VH and VL-domain contributions (see Methods). Regression lines, reported Pearson’s coefficients of correlation (*R*) and corresponding p-values (*p*) have been calculated by excluding the outlier point circled in red (mAb3).

We found a strong agreement between the calculated solubility score and the measured *PEG*_*1/2*_ (Figure 4). In particular, if one outlier point is excluded (mAb3, see below), the Pearson’s coefficient of correlation *R* between predictions and measurements is 0.97 (*p* < 10^−4^) when only the VH solubility score is considered, and 0.93 (*p* < 10^−3^) for the combined-chain solubility score. The inclusion of the outlier point brings the correlation down to *R* ~ 0.79 and 0.68 respectively (*p < *0.05). It is also worth noting that albeit mAb1, mAb4 and mAb8, as well as mAb7 and mAb9 are similar in terms of *PEG*_*1/*2_ solubility measurements, the sequences of the mAbs within these two groups are different (Figures 2 and S1). Therefore, the fact that the CamSol method correctly predicts similar solubility scores for these variants is by no means trivial.

The fact that most mutations between the different mAbs are actually found on the VH domain (Figure 2) likely explains the observation that the VH solubility score is a slightly better predictor of the observed differences in solubility than the combined VH-VL score (Figure 4). Indeed, the CamSol intrinsic solubility profiles of the VL domains are very similar among all variants (Figure S3), especially in the regions above the score of 1 (aggregation resistant) and below the score of −1 (aggregation promoting), which are particularly important in affecting self-association^19^. Conversely, the CamSol intrinsic solubility profiles of the VH domains show large differences in these regions, in particular close to the complementarity determining regions (CDR), where most mutations are found (Figure S3). Thus, it is possible that differences in hot-spot regions (i.e. the regions with scores above 1 and below −1) on the VH domain are key in determining the differences in the self-association behaviour, with the VL domain essentially not contributing to the observed solubility differences. In any case, with the exception of mAb3, both scores are highly effective in predicting the experimental outcome from the amino acid sequence alone.

We next asked whether the outlier mAb3 had any peculiar property that makes it difficult to predict its solubility in a reliable manner. First, as the measured solubility of mAb2 is very similar to that of mAb3 and it is correctly predicted, we compared the CamSol profiles of mAb3 and mAb2. The comparison between the intrinsic solubility profiles reveals that the higher predicted solubility of mAb3 predominantly comes from the CDR1 of the VH domain (Figure S4a). Within this loop, the most noticeable difference in the physicochemical properties of the amino acid sequences is the charged motif HDDS at position 26–29 of mAb3, which is found in place of GGTF in mAb2. Given that mAb3 and mAb2 have a similar measured solubility, these specific differences in the sequence are unexpected and difficult to interpret according to standard physicochemical rules. Mutations to charged residues resulting in increases in the absolute value of the net charge, which are associated with a higher predicted solubility of mAb3 with respect to that of mAb2, are in fact well-known to be strong factors that usually increase the solubility, as extensively reported in the literature^8, 19, 23, 28–30^. However, the combined VH/VL sequence of mAb3, when compared with those of the other mAbs, does not have a particularly high absolute net charge, despite having the highest fraction of charged residues (Figure S4b). Given this finding, we asked whether or not the self-association of mAb3 results from electrostatic attraction, such as dipole-dipole or higher-moment polar interactions, an effect that would not be captured by the sequence-based calculation performed by CamSol. However, the calculated dipole moment^31^ of the VH/VL domain of mAb3 is not considerably different from those of the other mAbs, and neither it points in a completely different direction (Figure S4c). Taken together these results do not provide a definite explanation for why mAb3 is predicted to be more soluble than it is actually measured to be. It may be that the CDR1 of the VH domain is not particularly important in affecting its self-association, or that the aggregation is indeed driven by electrostatic interactions that cannot be captured by a simple calculation of the dipole moment.

Finally, while this validation is performed on a small library of nine antibodies, the calculation of the intrinsic solubility score can be performed for thousands of sequences in a few seconds on a standard laptop. Therefore, the *in silico* solubility screening performed here is readily scalable to much larger libraries, for which performing exhaustive and accurate solubility measurements would be highly impractical. We thus anticipate that the library-screening strategy that we propose (Figure 1) will be very valuable in assisting lead selection in order to improve the chances of successful drug development.

### Aggregation hot-spot analysis and benchmark on mutational variants of mAb2

The antibody mAb2 was generated by *in vitro* affinity maturation of mAb1 via targeted and random mutagenesis using phage and ribosome display^22^, and it binds NGF with a *K*_*d*_ of 1.6–9.8 pM^20^. However, mAb2 has a strong tendency to self-associate, which results in very poor developability compared with mAb1 (Figure 3).

Recently the amino acids responsible for the aberrant self-interactions of mAb2 were identified using a structural proteomics approach^20^ (in ref. 20 mAb1 and mAb2 are named respectively MEDI578 and MEDI1912). The specific residues buried in the homodimer interface of mAb2 were revealed through a combination of hydrogen/deuterium exchange and cross-linking-mass spectometry^20^. Then, filtering for those amino acids that were actually different from mAb1, together with a selection of hydrophobic, solvent-exposed residues, narrowed the list down to three sites in the heavy chain (*Trp30, Phe31, Leu57*). These residues were systematically mutated back to their original types in mAb1 (respectively *Ser30*, *Thr31*, and *Thr57*), and the solubility of these variants was assessed through high-performance size-exclusion chromatography^20^.

**Figure 5:**
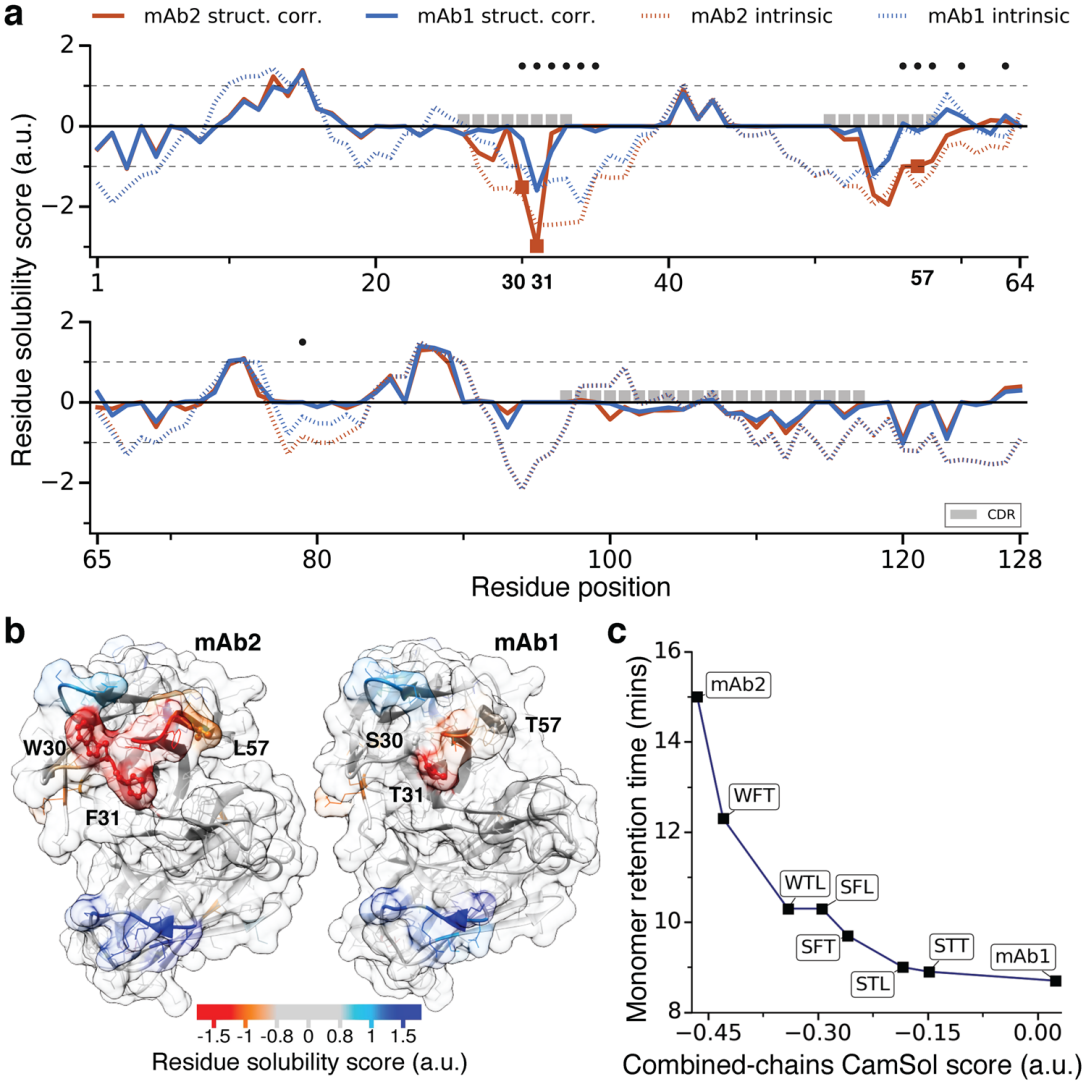
Aggregation hot-spot analysis and validation on mutational variants or mAb2. (**a**) Structurally-corrected (solid lines) and intrinsic (broken lines) CamSol solubility profiles for the VH domain of mAb1 (blue) and mAb2 (red). Residue positions at which the two sequences are different are labelled with a black dot above the profiles, CDR positions (IMGT annotation) with grey boxes. Square markers on the structurally corrected profile denote the positions on mAb2 (*W30, F31, L57*) that have been experimentally identified as aggregation hot-spots^20^. (**b**) The structurally corrected solubility profile is color-coded on the surface of homology models (built with SabPred^36^) of the VH/VL domains of mAb2 (left) and mAb1 (right). Aggregation-promoting regions are in orange/red while aggregation-protecting ones in light blue/blue. The experimentally-identified self-association hotspots on mAb2 and the corresponding residues on mAb1 are labelled and represented as balls-and-sticks. (**c**) Measured HP-SEC monomer retention time^20^ for various mAb variants as a function of their combined-chain solubility score calculated from the sequence alone. mAb2 has the residue types *W, F*, and *L* at the hot-spot positions 30, 31 and 57 respectively, while mAb1 has *S, T*, and *T*. The six variants between mAb2 and mAb1 are named according to which mAb2 position(s) have been mutated to the corresponding mAb1 amino acid^20^ (e.g. WFT is mAb2 L57T). The line is a guide for the eyes.

In order to identify aggregation hot-spots *in silico* we ran the CamSol structurally corrected algorithm^19^ on homology models of the variable domains of the two mAb variants. Strikingly, the residues of mAb2 with lowest structurally corrected solubility score, among those that differ between mAb1 and mAb2, are precisely those found experimentally (Figure 5a,b), with the addition of *Gly56* that was also detected by the structural-proteomic measurements but discarded from the list of candidate mutation sites as non-hydrophobic^20^.

Moreover, the sequence-based CamSol solubility score for each of the single, double, and triple mutants at the three identified sites is in perfect, albeit non-linear, agreement with measurements of monomer retention time performed with high-performance size-exclusion by the authors of ref. 20 (Figure 5c). The lack of linearity is likely due to the fact that measurements of monomer retention time tend to have poor resolution for highly soluble variants, and may exaggerate the differences among poorly soluble ones. For instance, above a certain solubility threshold, molecules of the same size will elute after the same time, while minor differences in the sticking to the column matrix may cause large differences in the observed monomer retention time.

The results of this analysis, together with those from previous applications of the CamSol method^19, 23, 32^, demonstrate that the calculation of the structurally-corrected solubility profile can effectively replace experiments for the mapping of aggregation-driving residues on the protein surface (Figure 5a,b). The fast solubility prediction embodied in the sequence-based intrinsic solubility score can then be used to reliably predict solubility-improving mutations at those sites, also in the case of pharmaceutically relevant monoclonal antibodies (Figure 5c).

## Discussion and Conclusions

In this study, we have used the CamSol method to quantitatively predict the solubility of a panel of monoclonal antibodies against NGF using only their amino acid sequences as input (Figure 4). We have further shown that a simple homology model can be exploited to map aggregation hotspots on the antibody surface using the CamSol structurally-corrected solubility profile, and how the impact of mutations at these sites on the solubility can be quantitatively predicted using intrinsic solubility scores.

While solubility-improving mutations on antibodies can be predicted with high accuracy (Figure5), as also demonstrated by previous applications of CamSol^19^ and of other methods^8, 33^, it remains challenging to incorporate the rational design of solubility as an integral part of an antibody development pipeline. The main problem is that aggregation-driving residues on antibody surfaces tend to be found close to, or within the CDR loops, which are also primarily responsible for antigen binding. Thus, predicting mutations that, besides improving the solubility, have no impact on the binding affinity or on the native-state stability requires a significant amount of information on the specific antibody under scrutiny. Structural information about the solvent-exposure, and relative distance of amino acids are readily obtained from homology models, which for antibodies can usually be computed with high-enough accuracy^34–36^. However, these models do not provide information about those specific residues that are part of the paratope. Likewise, it is often not enough to know which residues are actually in contact with the antigen in the bound structure, as nearby residues may stabilize the conformation of the binding loops and have a significant impact on the binding affinity. In the specific case of the analysis reported in Figure 5, mutation selection was aided by the fact that mAb2 and mAb1 were both known to bind NGF with good affinity, and also by the availability of a bound crystal structure of mAb1^20^. Indeed, selected mutations in mAb2 are to residues found in mAb1 and not involved in direct contact with the antigen^20^. This detailed knowledge of the antibody under scrutiny, however, is seldom available at the early stages of development.

By contrast, the sequence-based solubility screening that we have introduced here to rank libraries of protein variants (Figures 1 and 4) only requires as input the amino acid sequences of the antibodies under scrutiny and no further knowledge about them. Therefore, it can readily be used immediately after the sequencing of the screened library to help select antibodies with high solubility as well as binding affinity (Figure 1). We envisage that this type of screening will dramatically reduce the need for solubility measurements, as well as decrease the chances of failure of the selected lead antibodies in developability assessments. Since the CamSol method can run thousands of sequences in a few seconds on standard laptops, solubility predictions can readily be interlaced in the lead selection process from libraries of any size, and may also help the design of ‘smart’ libraries through comprehensive targeted mutagenesis of predicted aggregation-promoting regions, encompassing tens or hundreds of thousands mutations. As the CamSol method is available as a web server (www-mvsoftware.ch.cam.ac.uk), research groups that produce antibodies can readily apply it after library screening to aid the selection of the most promising variants.

## Material and Methods

### Monoclonal antibodies

All nine monoclonal antibodies (mAbs1-9) used in this study were supplied in 25 mM acetate, 240 mM sucrose, pH 5.0 by MedImmune (Cambridge, UK).

### CamSol version 2

To calculate the intrinsic solubility score for each of the mAbs we have used version 2 of the CamSol method^19^. This version was first introduced in 2015 for the proteome-wide analysis performed in ref. 37, and the main difference with version 1 is in the way the solubility score is calculated from the intrinsic solubility profile. Indeed, while version 1 was very powerful in predicting solubility changes up to a small number of mutations, its accuracy quickly decreased with increasing number of mutations. In the development of version 2 we thus focused on making the method more generally applicable in order to quantitatively rank the solubility of more distantly related proteins. The calculation of the intrinsic solubility profile (one number per amino acid in the input sequence) is described in details in ref. 19, while the solubility score for the protein *S*_*P*_ is now calculated from the profile using

1 $${S}_{P}=\frac{{\sum }_{i=1}^{N}\{\begin{array}{c}{\omega }_{up}({S}_{i}-t{h}_{up})\,if\,{S}_{i} > t{h}_{up}\\ {\omega }_{low}({S}_{i}-t{h}_{low})\,if\,{S}_{i} < t{h}_{low}\\ 0\qquad \quad \qquad \,otherwise\end{array}}{\gamma {N}^{\delta }}$$

where *S*_*i*_ is the value of the intrinsic solubility profile for the amino acid *i* and *N* the length of the input sequence. The upper and lower thresholds *th*_*up*_ and *th*_*low*_, as well as the coefficients ω_*up*_, ω_*low*_, *ɣ* and *δ* were fitted with a Monte Carlo procedure aimed at maximizing both the absolute value of the coefficient of correlation of *S*_*P*_ with measurements of aggregation rates from the literature^38^, and the ability of Sp to discriminate between non-aggregating and aggregating peptides and proteins collected through a systematic literature search by the authors of ref. 39 (file S2 therein), which contains totally unrelated sequences rather than mutational variants of the same protein. After the fitting of these parameters the performance of the new score was further tested by verifying that both the strong quantitative correlation with the solubility measurements reported in the original CamSol paper^19^ was not affected, and that the qualitative validation on solubility measurements from the literature^19^ still held (it changed from 54/56 to 55/56 correctly predicted solubility changes). Finally, since the scores computed with Eq. 1 are dimensionless numbers, they are rescaled so that the mean value and standard deviation calculated among more than 10^6^ random sequences are 0 and 1 respectively (S_p_ = [S_p_ − μ_random_]/σ_random_). Random sequences used in this calculation were generated with the same amino acid frequency and length distribution of the human proteome.

### Combined-chains CamSol score

The combined-chains CamSol score is calculated with Eq. 1 by using as input profile the joined intrinsic solubility profiles of the different amino-acid chains (in the case of mAbs the heavy and light chains), so that *N* becomes the total number of amino acids in the complex. Note that the order in which the profiles of the chains are joined together does not affect the resulting solubility score.

### Chemicals and consumables

PEG 8000 was purchased from Alfa Aesar. Sodium acetate, sodium citrate and sodium phosphate were purchased from JT Baker. Acetic acid was purchased from VWR and sucrose was purchased from Sigma. UV-Star™ µClear™ 96-well microplates (from Greiner Bio-One) were used for both sample preparation and collection of filtrate. FiltrEX™ 96-well filter plates with 0.2 µm PVDF membrane (Corning,) were used for sample filtration. Microseal® ‘B’ Adhesive Sealing Film (from BIO-RAD) was used for sealing plates during incubation. Droplate96-D+ chips (Perkin Elmer,) were used for measuring absorbance at 280 nm the LabChip DS (by Perkin Elmer, Waltham, MA).

### 40% (w/v) PEG 8000 stock solutions

When dissolving PEG in buffered solutions a shift was observed in measured pH. Therefore, in order to achieve the target pHs, stock solutions of 40% (w/v) PEG 8000 were prepared in both 10 mM citrate and 10 mM phosphate buffer. These solutions were then used to generate the final 40% (w/v) PEG 8000 stocks in 10 mM citrate/phosphate buffer at pH 5.5.

### 10 mM citrate/phosphate buffer stock solutions

Stock solutions of 10 mM citrate and 10 mM phosphate buffer were titrated to attain final 10 mM citrate/phosphate stock solutions at pH 5.5.

### mAb stock solutions

All nine mAbs were diluted with 25 mM acetate, 240 mM sucrose, pH 5.0 to a target final concentration of 10 mg/mL.

### PEG-precipitation assay

#### Plate preparation

For measurements at pH 5.5 differing ratios of 10 mM citrate/phosphate, pH 5.5 and 40% (w/v) PEG 8000 in 10 mM citrate/phosphate, pH 5.5, were combined with 20 µL of mAb stock solution to achieve a PEG concentration range of 0–16% (w/v) and final mAb concentration of 1 mg/mL. A further 0–8% (w/v) PEG dilution series was used for mAbs with lower solubility as inferred from the 0–16% (w/v) range. The resulting mAb/PEG samples were prepared and assayed in triplicate. *Plate incubation and analysis:* Following preparation, plates were sealed with Adhesive Sealing Film and incubated at 2–8 °C for 48 hours. After incubation, samples were thoroughly pipette mixed in their respective wells and filtered using 0.2 µm filter plates. The collected filtered samples were mixed again before transferring 2 µL of each sample to a LabChip DS plate for protein concentration determination at 280 nm on a LabChip DS (by Perkin Elmer, Waltham, MA) using the respective theoretical extinction coefficient for each mAb.

#### Data fitting

data points in Figure 3 were fitted with the equation

$$y=\frac{a}{1+{e}^{s(x-PE{G}_{1/2})}}+b$$

where the free parameters are the sigmoid slope *s* and the midpoint *PEG*_*1/2*_. The parameters *a* and *b* have been introduced to better fit the data in order to obtain a more accurate estimate of *PEG*_*1/2*_ and were only allowed to vary in (0.9,1.1) and (0,0.1) respectively, while *a* was fixed to 1 for the least soluble antibodies (mAb2, 3 and 6), where a clear plateau at low PEG concentrations is not observed.

### Differential scanning calorimetry (DSC)

Protein unfolding was assessed in 10 mM citrate/phosphate, pH 5.5 by differential scanning calorimetry. Measurements were conducted on a Nano DSC (TA Instruments) in a 96-well format. mAbs1-9 at 10 mg/mL in 25 mM acetate, 240 mM sucrose, pH 5.0 were diluted to 1 mg/mL with 10 mM citrate/phosphate, pH 5.5. Samples were stored at 5 °C in a 96-well plate then heated from 25 °C to 100 °C at 95 °C/hr. The thermograms were background corrected, normalized and fitted using a 2-state scaled model to determine thermal melt temperatures.

### Differential scanning fluorimetry (DSF)

Conformational stability was assessed for triplicate samples in 10 mM citrate/phosphate, pH 5.5 by differential scanning fluorimetry. Measurements were conducted on a CFX96 RT-PCR system (BIO-RAD) in a 96-well format combining mAbs 1–9 at 10 mg/mL in 25 mM acetate, 240 mM sucrose, pH 5.0 with 10 mM citrate/phosphate, pH 5.5 and SYPRO orange fluorophor (Invitrogen) to give a final concentration of 1 mg/mL. Samples were heated from 20 °C to 95 °C at 0.2 °C/min. Melt curves were generated automatically in CFX Manager (BIO-RAD) software with fluorescence intensities plotted as a function of temperature and melt temperatures determined.

## Electronic supplementary material

Supplementary Information
