# Supplementary material for: Rapid and accurate in silico solubility screening of a monoclonal antibody library
Source: Sci Rep. 2017 Aug 15;7:8200. doi: 10.1038/s41598-017-07800-w (PMC5558012; doi:10.1038/s41598-017-07800-w)
Supplement: Supplementary file 1 — Supplementary Information [file 41598_2017_7800_MOESM1_ESM.pdf]

Rapid and accurate *in silico* solubility screening of a monoclonal antibody library

Pietro Sormanni[1], Leanne Amery[2], Sofia Ekizoglou[2],
Michele Vendruscolo[1*] and Bojana Popovic[3*]

[1]*Department of Chemistry, University of Cambridge, Cambridge CB2 1EW, UK*
[2] *Biopharmaceutical Development, Medimmune Ltd, Granta Park, Cambridge, CB21 6GH, UK*
[3] *Antibody Discovery and Protein Engineering, Medimmune Ltd, Granta Park, Cambridge, CB21 6GH, UK*

**Corresponding authors: mv245@cam.ac.uk (MV), PopovicB@medimmune.com (BP)*

VH sequences

	1	11	21	31	41
mAb1	QVQLVQSGAE	VKKPGSSVKV	SCKASGGTFS	TYGISWVRQA	PGQGLEWMGG
mAb2	QVQLVQSGAE	VKKPGSSVKV	SCKASGGTFW	FGAFTWVRQA	PGQGLEWMGG
mAb3	QVQLVQSGAE	VKKPGSSVKV	SCKASHDDSI	LYGISWVRQA	PGQGLEWMGG
mAb4	EVQLVQSGAE	VKKPGSSVKV	SCKASGGTFS	TYGISWVRQA	PGQGLEWIGG
mAb5	EVQLVQSGAE	VKKPGSSVKV	SCKASGGTFS	TYGISWVRQA	PGQGLEWIGG
mAb6	QVQLVQSGAE	VKKPGSSVKV	SCKASWADMV	FYGISWVRQA	PGQGLEWMGG
mAb7	QVQLVQSGAE	AKKPGSSVKV	SCKASGGTFS	TYGISWVRQA	PGQGLEWMGG
mAb8	QVQLVQSGAE	VKKPGSSVKV	SCKASGGTFS	TYGISWVRQA	PGQGLEWMGG
mAb9	EVQLVQTGAE	VKKPGSSVKV	SCKASGGTFS	TYGISWVRQA	PGQGLEWIGG

	51	61	71	81	91
mAb1	IIPIFDTGNS	AQSFQGRVTI	TADESTSTAY	MELSSLRSED	TAVYYCARSS
mAb2	IIPIFGLTNL	AQNFQGRVTI	TADESTSTVY	MELSSLRSED	TAVYYCARSS
mAb3	VIPIFNLAHE	AQGFQGRVTI	TADESTSTAY	MELSSLRSED	TAVYYCARSS
mAb4	IIPIFDTGNS	AQSFQGRVTI	TADESTSTAY	MEVSSLRSDD	TAVYYCASSS
mAb5	IIPIFDAGNS	AQSFQGRVTI	TADESTSTAH	MEVSSLRSED	TAVYYCASSS
mAb6	IIPIFDTGNS	AQSFQGRVTI	TADESTSTAY	MELSSLRSED	TAVYYCTASP
mAb7	IIPIFDTGNS	ARSFQGRVTI	TADESTSTAY	MELSSLRSED	TAVYYCASSS
mAb8	IIPIFDTGNS	AQSFQGRVTI	TADESTSTAY	MELSSLRSED	TAVYYCAASN
mAb9	IIPIFDTGNS	AQSFQGRVTI	TADESTSTAY	MEVSSLRSDD	TAVYYCASSS

	101	111	121
mAb1	RIYDLNPSLT	AYYDMDVWGQ	GTMVTVSS
mAb2	RIYDLNPSLT	AYYDMDVWGQ	GTMVTVSS
mAb3	RIYDLNPSLT	AYYDMDVWGQ	GTMVTVSS
mAb4	RIYDYAGGDH	YYYDMDVWGQ	GTMVTVSS
mAb5	RIYDHHIQKG	GYYDMDVWGQ	GTMVTVSS
mAb6	RLYDLNPSLT	AYYDMDVWGQ	GTMVTVSS
mAb7	RIYDLNPSLT	AYYDMDVWGQ	GTMVTVSS
mAb8	KLYDLNPSLT	AYYDMDVWGQ	GTMVTVSS
mAb9	RIYDANRQAV	PYYDMDVWGQ	GTMVTVSS

VL sequences

	1	11	21	31	41
mAb1	QSVLTQPPSV	SAAPGQKVTI	SCSGSSSNIG	NNYVSWYQQL	PGTAPKLLIY
mAb2	QSVLTQPPSV	SAAPGQKVTI	SCSGSSSDIG	NNYVSWYQQL	PGTAPKLLIY
mAb3	QSVLTQPPSV	SAAPGQKVTI	SCSRSSSNIG	NNYVSWYQQL	PGTAPKLLIY
mAb4	QAVLTQPSSV	STPPGQKVTI	SCSGSSSNIG	NNYVSWYQQL	PGTAPKLLIY
mAb5	QAVLTQPSSV	STPPGQKVTI	SCSGSSSNIG	NNYVSWYQQL	PGTAPKLLIY
mAb6	QSVLTQPPSV	SAAPGQKVTI	SCSGSSSNIG	NNYVSWYQQL	PGTAPKLLIY
mAb7	QSVLTQPPSV	SAAPGQKVTI	SCSGSSSDIG	NNYVSWYQQL	PGTAPKLLIY
mAb8	QSVLTQPPSV	SAAPGQKVTI	SCSGSSSDVG	NNYVSWYQQL	PGTAPKLLIY
mAb9	QAVLTQPSSV	STPPGQMVTI	SCSGSSSDIG	NNYVSWYQQL	PGTAPKLLIY

	51	61	71	81	91
mAb1	DNNKRPSGIP	DRFSGSKSGT	SATLGITGLQ	TGDEADYYCG	TWDSSLSAWV
mAb2	DNNKRPSGIP	DRFSGSKSGT	SATLGITGLQ	TGDEADYYCG	TWDSSLSAWV
mAb3	DNNKRPSGIP	DRFSGSKSGT	SATLGITGLQ	TGDEADYYCG	TWDSSLSAWV
mAb4	DNNKRPSGIP	DRFSGSKSGT	SATLGITGLQ	TGDEADYYCG	TWDSSLSAWV
mAb5	DNNKRPSGIP	DRFSGSKSGT	SATLGITGLQ	TGDEADYYCG	TWDSSLSAWV
mAb6	DNNKRPSGIP	DRFSGSKSGT	SATLGITGLQ	TGDEADYYCG	TWDSSLSAWV
mAb7	DNNKRPSGIP	DRFSGSKSGT	SATLGITGLR	TGDEADYYCG	TWDSSLSAWV
mAb8	DNNKRPSGIP	DRFSGSKSGT	SATLGITGLQ	TGDEADYYCG	TWDSSLSAWV
mAb9	DNNKRPSGIP	DRFSGSKSGT	SATLGITGLQ	TGDEADYYCG	TWDSSLSAWV

	101
mAb1	FGGGTKLTVL
mAb2	FGGGTKLTVL
mAb3	FGGGTKLTVL
mAb4	FGGGTKLTVL
mAb5	FGGGTKLTVL
mAb6	FGGGTKLTVL
mAb7	FGGGTKLTVL
mAb8	FGGGTQLTVL
mAb9	FGGGTKLTVL

Figure S1. Multiple sequence alignment of the sequences of the VH domains (top) and VL domains (bottom) of the mAb variants used in this study. Residues are coloured according to the Clustal-X standard colour scheme. These sequences are the input for the CamSol intrinsic solubility calculations performed in this study.

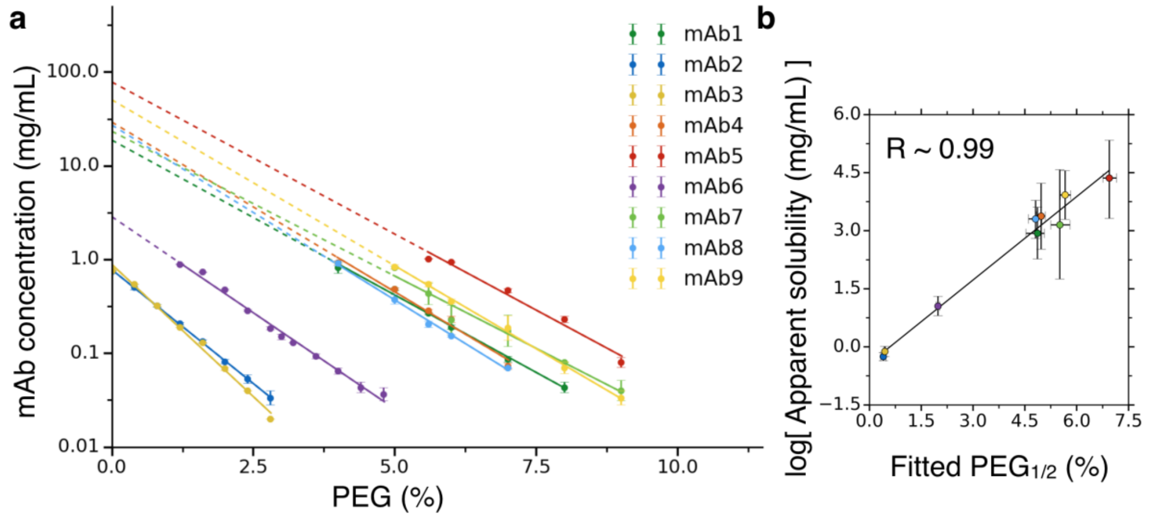

Figure S2 (**a**) Plot of mAb concentrations (y-axis; log-scale) versus weight-*% PEG* amount (x-axis). The concentration value at *%PEG=0* (the y-intercept) extrapolated from the linear fitting is referred to as apparent solubility[1], and it is reported on the y-axis of the scatter plot in panel (**b**) as a function of the $PEG_{1/2}$ parameter from the fitting of the sigmoid from **Figure 3**. Error bars in (**b**) are 95% confidence interval on the fitting parameters calculated with 10^4 bootstrap cycles (**Table 1**).

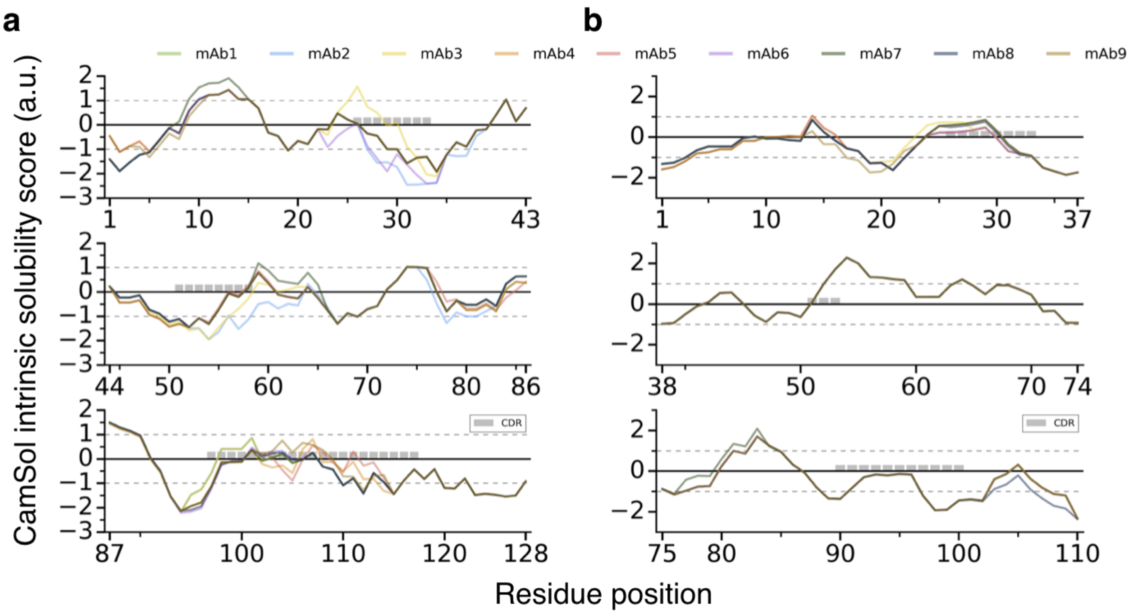

Figure S3. CamSol intrinsic solubility profiles of the nine mAbs studied in this work. Plot of the intrinsic solubility profiles calculated with the CamSol method from the sequences of the VH (**a**) and VL (**b**) domains of the mAbs used in this study (legend). Residue-positions corresponding to CDR loops according to the IMGT annotation scheme are marked with grey boxes along the x-axis.

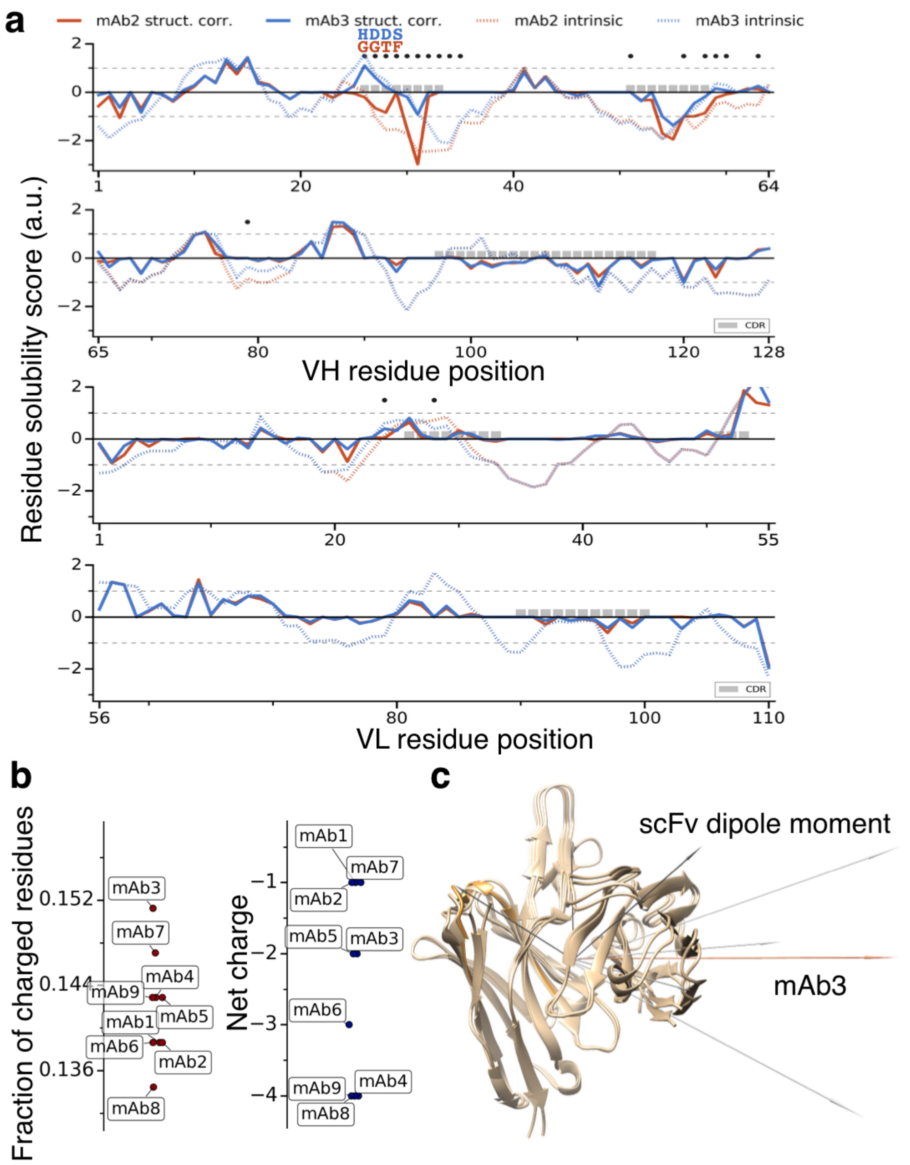

Figure S4. Analysis of the physicochemical properties of mAb3. Comparison of the structurally-corrected (solid lines) and intrinsic (broken lines) CamSol solubility profiles for the VH (top plot) and VL (lower plot) domains of mAb3 (blue) and mAb2 (red). CDR positions are depicted with grey boxes (IMGT annotation). Residue positions at which the two sequences are different are labelled with a black dot above the profiles; the first four amino acids of the CDR1 are written above the dots in one-letter notation. (**b**) Distributions of the fraction of charged residues (left) and net charge (right) of the combined VH/VL sequence of the nine mAbs. (**c**) Superimposed homology models (built with SabPred[2]) of the VH/VL domains of all nine mAbs with their calculated dipole moments[3] depicted as arrows of length proportional to the dipole intensity; the dipole moment of mAb3 is coloured in orange.

Supplementary references

1. Atha, D. H. & Ingham, K. C. Mechanism of precipitation of proteins by polyethylene glycols. Analysis in terms of excluded volume. *J. Biol. Chem.* **256,** 12108–12117 (1981).
2. Dunbar, J. *et al.* SAbPred: a structure-based antibody prediction server. *Nucleic Acids Res.* **44,** W474–8 (2016).
3. Felder, C. E., Prilusky, J., Silman, I. & Sussman, J. L. A server and database for dipole moments of proteins. *Nucleic Acids Res.* **35,** W512–21 (2007).
